# USING SOCIAL CONVOY THEORY TO IDENTIFY AND RECRUIT CAREGIVERS FOR HEALTH-RELATED AGING RESEARCH

**DOI:** 10.1093/geroni/igae098.2425

**Published:** 2024-12-31

**Authors:** Jennifer Portz, Josh Silvasstar, Heather Coats

**Affiliations:** University of Colorado, Aurora, Colorado, United States; Colorado School of Public Health, Aurora, Colorado, United States; University of Colorado Anschutz Medical Campus, Aurora, Colorado, United States

## Abstract

This study focuses on addressing the challenge of identifying and recruiting caregivers for health-related aging research. Social Convoy Theory, a framework for capturing social support over the lifespan, may be a helpful recruitment model. As part of a larger qualitative project, the objective is to compare two recruitment

**methods:**

the “Helpers” Strategy, which directly asks older adults about who “helps” them with their health, and the “Social Convoy” Strategy, which utilizes a Social Convoy approach to complete a social support diagram. Both strategies involved contacting identified caregivers by phone, email, and text for recruitment. Analysis of the two strategies leveraged a data visualization tool, Tableau, to examine the data. Both strategies successfully identified at least one caregiver among 77-78% of the 31 older adults enrolled in the qualitative study, resulting in a total of 49 caregivers. However, the Social Convoy Strategy yielded more caregivers per participant (1.72) compared to the Helpers Strategy (1.5). While the Helpers Strategy predominantly identified one caregiver, the Social Convoy Strategy identified a broader range, including convoys of multiple caregivers. Caregiver participation rate was 61.2%, with the Helper Strategy showing a higher rate of participation (66.7%) than the Social Convoy Strategy (58.1%). However, the Social Convoy Strategy facilitated interviews with more caregivers across the network. The findings suggest that although the Social Convoy Strategy may have a lower overall recruitment rate, it enables the identification and interview participation of more than one caregiver per older adult across the caregiving network compared to the Helper Strategy.

